# Papillary Muscle Infarction by Cardiac MRI in Patients With Mitral Regurgitation

**DOI:** 10.1002/clc.24312

**Published:** 2024-07-02

**Authors:** Jie Hou, Yu Sun, Huishan Wang, Libo Zhang, Benqiang Yang

**Affiliations:** ^1^ College of Medicine and Biological Information Engineering Northeastern University Shenyang Liaoning China; ^2^ Department of Radiology General Hospital of Northern Theater Command Shenyang China; ^3^ Key Laboratory of Cardiovascular Imaging and Research of Liaoning Province Shenyang China; ^4^ Department of Cardiovascular Surgery General Hospital of Northern Theater Command Shenyang China

**Keywords:** arrhythmia, mitral regurgitation, papillary muscle, papillary muscle infarction

## Abstract

**Background:**

Papillary muscle (PM) infarction (PMI) detected by cardiac magnetic resonance imaging (CMR) is associated with poor outcomes. Whether PM parameters provide more value for mitral regurgitation (MR) management currently remains unclear. Therefore, we examined the prognostic value of PMI using CMR in patients with MR.

**Methods:**

Between March 2018 and July 2023, we retrospectively enrolled 397 patients with MR undergoing CMR. CMR was used to detect PMI qualitatively and quantitively. We also collected baseline clinical, echocardiography, and follow‐up data.

**Results:**

Of the 397 patients with MR (52.4 ± 13.9 years), 117 (29.5%) were assigned to the PMI group, with 280 (70.5%) in the non‐PMI group. PMI was demonstrated more in the posteromedial PM (PM‐PM, 98/117) than in the anterolateral PM (AL‐PM, 45/117). Compared with patients without PMI, patients with PMI had a decreased AL‐PM (41.5 ± 5.4 vs. 45.6 ± 5.3)/PM‐PM diastolic length (35.0 ± 5.2 vs. 37.9 ± 4.0), PM‐longitudinal strain (LS, 20.4 ± 6.1 vs. 24.9 ± 4.6), AL‐PM‐LS (19.7 ± 6.8 vs. 24.7 ± 5.6)/PM‐PM‐LS (21.2 ± 7.9 vs. 25.2 ± 6.0), and increased inter‐PM distance (25.7 ± 8.0 vs. 22.7 ± 6.2, all *p* < 0.001). Multiple logistic regression analyses identified male sex (odds ratio [OR] = 3.65, 95% confidence interval = 1.881−7.081, p < 0.001) diabetes mellitus (OR/95% CI/*p* = 2.534/1.13–5.68/0.024), AL‐PM diastolic length (OR/95% CI/*p* = 0.841/0.77–0.92/< 0.001), PM‐PM diastolic length (OR/95% CI/*p* = 0.873/0.79–0.964/0.007), inter‐PM distance (OR/95% CI/*p* = 1.087/1.028–1.15/0.003), AL‐PM‐LS (OR/95% CI/*p* = 0.892/0.843–0.94/< 0.001), and PM‐PM‐LS (OR/95% CI/*p* = 0.95/0.9–0.992/0.021) as independently associated with PMI. Over a 769 ± 367‐day follow‐up, 100 (25.2%) patients had arrhythmia. Cox regression analyses indicated that PMI (hazard ratio [HR]/95% CI/*p* = 1.644/1.062–2.547/0.026), AL‐PM‐LS (HR/95% CI/*p* = 0.937/0.903–0.973/0.001), and PM‐PM‐LS (HR/95% CI/*p* = 0.933/0.902–0.965/< 0.001) remained independently associated with MR.

**Conclusions:**

The CMR‐derived PMI and LS parameters improve the evaluation of PM dysfunction, indicating a high risk for arrhythmia, and provide additive risk stratification for patients with MR.

## Graphical abstract

Papillary muscle infarction: diagnosis, assessment, and prognosis. The present analysis is based on CMR to qualitatively and quantitatively assess the papillary muscle infarction (PMI) and longitudinal strain of anterolateral (AL‐PM‐LS) and posteromedial papillary muscle (PM‐PM‐LS) in patients with MR. PMI and PM strain parameters showed a high risk for follow‐up arrhythmias.

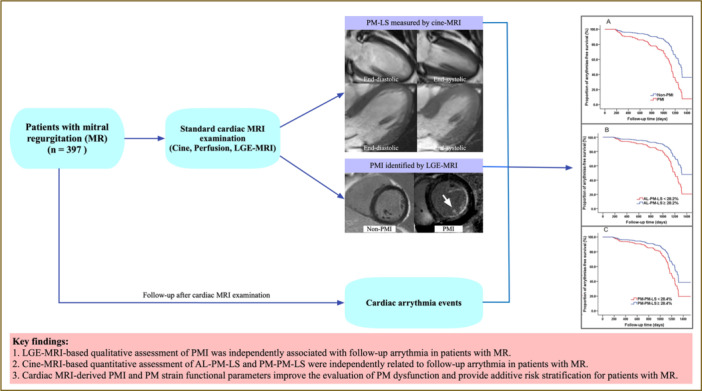

**Abbreviations**
AL‐PM: anterolateral papillary muscle
BMI: body mass index
CHD: coronary heart disease
CI: confidence interval
CK: creatine kinase
CK‐MB: creatine kinase‐MB
CMR: cardiac magnetic resonance imaging
EACTS: European Association for Cardio‐Thoracic Surgery
ECG: electrocardiography
ESC: European Society of Cardiology
hcrp: high‐sensitivity C‐reactive protein
HDL: high‐density lipoprotein
HR: hazard ratio
hsTNT: high‐sensitivity troponin T
ICC: intraclass correlation coefficients
LADD: left atrial end‐diastolic dimension
LDH: lactic dehydrogenase
LDL: low‐density lipoprotein
LGE: late gadolinium enhancement
LS: longitudinal strain
LVEDD: left ventricular end‐diastolic dimension
LVEDV: left ventricular end‐diastolic volume
LVEF: left ventricular ejection fraction
LVESV: left ventricular end‐systolic volume
LVSV: left ventricular stroke volume
MR: mitral regurgitation
myo: myoglobin
NT‐BNP: brain natriuretic peptide
NYHA: New York Heart Association
OR: odds ratio
PCI: percutaneous coronary intervention
PM: papillary muscle
PMI: papillary muscle infarction
PM‐PM: posteromedial papillary muscle
SD: standard deviation
TIA: transient ischemic attack

## 1 Introduction

Left ventricular papillary muscles (PMs) are small myocardial structures and play a significant role in the functioning of the mitral valve (MV) and the left ventricle (LV) [1]. During systole, the PM contracts before the LV wall contracts, causing apposition of the MV leaflets and limiting the retrograde blood flow from the LV back into the left atrium. PM dysfunction may result in mitral regurgitation (MR) [2].

PM infarction (PMI) is related to LV remodeling, increased mitral annular dilatation, systolic retraction of the mitral leaflets, and reduced coaptation, and development of MR is possibly mediated through infarction (lateral wall) and LV remodeling with secondary dysfunction of the MV apparatus [3, 4, 5, 6]. PMI is associated with impaired LV function and poor outcomes [3, 4] and could be related to ventricular arrhythmias [7, 8].

Cardiac magnetic resonance imaging (CMR) is a major imaging technique to evaluate the PM, providing information on morphology, function, and tissue characterization [9, 10]. Late gadolinium enhancement (LGE) imaging has emerged as the gold standard imaging modality to identify even small myocardial necrosis in vivo [11].

PMI with MR is associated with cardiac morbidity and mortality and is therefore considered to be an unfavorable prognostic factor [6, 12, 13]. Although PM viability is regarded as a prognostic factor when considering MV replacement or repair, the application value of qualitative and quantitative detection of PMI based on CMR is still uncertain. However, currently, there are no reports on the quantitative evaluation of PMI using CMR and exploration of its value in the diagnosis and treatment of MR diseases. The purpose of this study is to analyze the presence, proportion, and parameters including strain parameters of PMI depicted by CMR to assess its impact on patients with MR and follow‐up arrhythmias.

## 2 Materials and Methods

After obtaining approval from our ethics committee, informed patient consent was waived due to the retrospective nature of our investigation.

### 2.1 Patient Enrollment

This is a single‐center, retrospective, observational cohort study. Patients diagnosed with MR who underwent an examination with CMR from March 2018 to July 2023 were selected continuously. The baseline clinical characteristics, echocardiography, and CMR data were retrospectively collected. We adhered to 2021 European Society of Cardiology (ESC)/European Association for Cardio‐Thoracic Surgery (EACTS) guidelines outlining MR patient management [14]. Upon admission, patients underwent echocardiography and electrocardiography (ECG)/dynamic ECG [15].

The inclusion criteria were as follows: (1) patients with various degrees of MR, (2) patients who received CMR and current echocardiography detection, and (3) patients aged between 18 and 80 years. The exclusion criteria were as follows: (1) patients with congenital heart disease, (2) patients who had undergone previous valvular surgery or ablation for atrial fibrillation, (3) patients with general contraindications for MRI, and (4) patients with incomplete essential data and poor image quality.

### 2.2 Imaging

Imaging was performed on a 3.0 T MRI scanner (Verio, Siemens Medical Systems, Erlangen, Germany) using a 32‐channel phased‐array body coil. The CMR protocol included cine True Fast imaging with Steady‐State Precession (TrueFISP) imaging sequences in the short‐axis views and longitudinal two‐, three‐, and four‐chamber views covering the entire LV and LA. All images were ECG‐gated; patients were placed in the supine position and required to hold their breath during image capture. The sequence parameters were as follows: repetition time 40.68 ms, echo time 1.49 ms, field of view 360 × 360 mm, slice thickness 6 mm with no gap, flip angle 50°, and matrix size 216 × 256. In addition, we used the cardiac shim model of SIEMENS to adapt adjustment volume to reduce dark band artifacts. LGE images were acquired 10 min after administration of a 0.5‐Gd–based contrast agent with a dosage of 0.2 mmol/kg in the same short‐axis and para‐axial views.

### 2.3 Imaging Analysis

Images were analyzed using CVI42 (Version 5.12.1, Circle, Calgary, Canada).

PMIs were identified on short‐axis LGE CMR images, and the myocardium of PM was defined as infarcted on LGE CMR [8, 16]. PM involvement was assessed regarding the extent and location, and PMI was divided into anterolateral (AL)‐ and posteromedial (PM)‐PMI, as well as complete or partial infarction.

**Figure 1:**
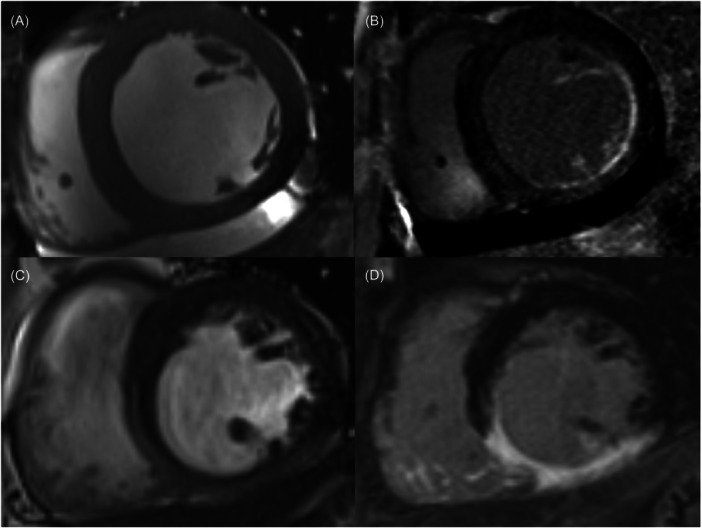
Assessment of PMI by CMR. (A, B) Patient with partial infarction of anterolateral PM and complete infarction of the posteromedial PM. (A) Cine image. (B) LGE image of the same location as a side‐by‐side reference for localizing the PM. (C, D) Patient with partial infarction of the posteromedian PM. (C) Cine image. (D) LGE image.

Complete PMI was indicated by hyperenhancement of the entire cross‐sectional area of the involved PM on LGE images, while partial PMI was indicated by hyperenhancement of a part of the cross‐sectional area of the involved PM on LGE images [14, 16, 17]. If PM enhancement was seen on images with LGE, PMI was considered (Figure 1).

PM‐longitudinal strain (PM‐LS) values have been assessed on cine MR images as the percentage of shortening between end‐diastolic and end‐systolic phases. PM‐LS = (end‐diastolic PM length − end‐systolic PM length)/end‐diastolic PM length × 100% [4]. MR images were separately and independently assessed by two independent experienced readers for the presence, extent, and location of PMI (J.H., 6 years of CMR experience; Y.S., 4 years of CMR experience). Discordance was settled by discussion and consensus. Quantitative assessment was performed by a radiologist (J.H.) who was unaware of the patients' clinical data and grouping information. The intra‐ and interobserver variabilities for the PM parameters and measurements were assessed by the intraclass correlation coefficient (ICC). Intraobserver reproducibility was established by the same observer (J.H.), who reanalyzed the same subjects after 1 month. Interobserver reproducibility was assessed by a second independent observer (Y.S.) who was blinded to the first observer's results.

### 2.4 Patient Follow‐Up

Follow‐up information was collected from outpatient visits. Patients were followed up to March 2024. Primary study outcomes were arrhythmia occurrence or recurrence during routine follow‐up (> 3 months after treatment), which required direct or drug current cardioversion. Follow‐up at outpatient visits or rehospitalization included echocardiography and ECG evaluations after treatment.

### 2.5 Statistical Analyses

Data were analyzed in SPSS version 20.0 (SPSS Statistics, IBM Corporation, Armonk, NY, USA). Continuous data were represented as the mean ± standard deviation (SD) or median (quartiles), and analyzed using independent‐sample *t*‐ or Mann–Whitney *U*‐tests. Normal distributions across continuous variables were examined using Kolmogorov–Smirnov tests. Pearson's *χ* ^2^ test or Fisher's exact test was used to assess categorical data (Table 1). Inter‐ and intraobserver agreement data for subjectively assessing PMI occurrence were evaluated using cross‐tabulation and kappa (*κ*) calculations. Moreover, ICCs were also used to assess the accuracy and precision of the method to measure each PM parameter. To determine significant independent predictors, multivariate logistic regression analyses were performed (Table 2). Also, multicollinearity analyses were performed to determine collinear covariates. We used Cox regression for follow‐up arrhythmia analyses, and parameters with significant effects in univariate Cox regression analysis were subjected to multivariate Cox regression (Table 3). We used receiver‐operating characteristic (ROC) analyses to examine predictive potential factors in a multivariate‐adjusted logistic regression model. The optimal cutoff point was identified using the Youden index at the maximum sum of sensitivity and specificity −1. The diagnostic accuracy was assessed using the area under the curve (AUC). Kaplan–Meier survival estimates were generated for patient groups stratified by the optimal cutoff point, along with a log‐rank test. A *p* < 0.05 value indicated statistical significance.

**Table 1:** Characteristics and clinical data of the study population.

Variables	Total (*n* = 397)	PMI (*n* = 117)	Non‐PMI (*n* = 280)	*p*
Age (years, mean ± SD)	52.4 ± 13.9	52.4 ± 14.3	52.3 ± 13.8	0.965
Sex (male, *n*, %)	245 (61.7)	87 (74.4)	158 (56.4)	0.001*
BMI (kg/m^2^)	25.1 ± 4.1	25.2 ± 3.5	25.1 ± 4.3	0.803
HR (bpm)	79.5 ± 14.9	78.9 ± 14.4	79.7 ± 15.1	0.634
Hypertension (*n*, %)	115 (29)	35 (29.9)	80 (28.6)	0.788
Diastolic pressure	131.9 ± 27.8	131.3 ± 26.4	132.1 ± 28.5	0.788
Systolic pressure	84.1 ± 16.4	84.5 ± 15.2	83.9 ± 16.8	0.742
Diabetes mellitus (*n*, %)	47 (11.8)	24 (20.5)	23 (8.2)	0.001*
Smoking (*n*, %)	107 (27)	42 (35.9)	65 (23.2)	0.009*
Alcohol (*n*, %)	87 (21.9)	32 (27.4)	55 (19.6)	0.091
NYHA class (> Ⅲ) (*n*, %)	199 (50.1)	58 (49.6)	141 (50.4)	0.887
History of TIA/infarction	20 (5)	7 (6)	13 (4.6)	0.578
CHD (*n*, %)	81 (20.4)	33 (28.2)	48 (17.1)	0.013*
Laboratory findings				
CK (U/L, median [IQR])	79 (54–112.5)	98 (60–129)	73 (53–103)	0.001*
CK‐MB (U/L)	19 ± 46	23 ± 64	17 ± 36	0.251
myo (mg/L)	36.2 (25.5–47)	40 (27.75–51.5)	35 (24–45)	0.003*
LDH (mg/L)	186 (157–224)	195 (168–230)	184 (154–217.5)	0.029*
hcrp (mg/L)	7.6 ± 19.4	9.8 ± 23.6	6.6 ± 17.4	0.14
HCY (mg/L)	12 (9.7–15)	12.7 (10–17.5)	11.8 (9.6–14.3)	0.005*
HDL (μmol/L)	1.12 ± 0.29	1.12 ± 0.25	1.13 ± 0.30	0.884
LDL (μmol/L)	2.7 ± 0.8	2.6 ± 0.9	2.7 ± 0.8	0.347
hsTNT (mg/L)	8 (0.03–17)	10 (0.04–25)	7 (0.01–15)	0.013*
NTProBNP (pg/mL)	802 (204–1795)	983.5 (265–2520)	684 (178–1632.5)	0.021*
Medication				
Aspirin (*n*, %)	22 (5.5)	9 (7.7)	13 (4.6)	0.226
Clopidogrel (*n*, %)	11 (2.8)	6 (5.1)	5 (1.8)	0.13
Statins (*n*, %)	53 (13.4)	17 (14.5)	36 (12.9)	0.655
Others	133 (33.5)	40 (34.2)	93 (33.2)	0.851
Etiology of MR				
Rheumatic heart disease	29 (7.3)	3 (2.6)	26 (9.3)	0.019*
Degenerative heart disease	187 (47.1)	14 (12.0)	173 (61.8)	< 0.001*
Ischemia/infarction‐related	181 (45.6)	100 (85.4)	81 (28.9)	< 0.001*
Management				
PCI	17 (4.3)	13 (11.1)	4 (1.4)	< 0.001*
MV repair	10 (2.5)	1 (0.9)	9 (3.2)	0.309
MV replacement	48 (12.1)	10 (8.5)	38 (13.6)	0.162
Aortic valve replacement	28 (7.0)	10 (8.5)	18 (6.4)	0.452
Tricuspid valve repair	31 (7.8)	7 (6.0)	24 (8.6)	0.381
CABG	2 (0.5)	1 (0.9)	1 (0.3)	0.503
Maze procedure	11 (2.8)	3 (2.6)	8 (2.9)	1.000
Others	250 (63.0)	72 (61.5)	178 (63.6)	0.702
CMR data				
PMI distribution				
Posteromedian PMI (*n*, %)	98 (24.7)	98 (83.8)	—	—
Incomplete (*n*, %)	89 (22.4)	89 (76.1)	—	—
Complete (*n*, %)	9 (2.3)	9 (7.7)	—	—
Anterolateral PMI (*n*, %)	45 (11.3)	45 (38.5)	—	—
Incomplete (*n*, %)	41 (10.3)	41 (35)	—	—
Complete (*n*, %)	4 (1)	4 (3.4)	—	—
Combined PMI (*n*, %)	22 (5.5)	22 (18.8)	—	—
Length and longitudinal strain values of PM				
AL‐PM diastolic length (mm)	44.4 ± 5.7	41.5 ± 5.4	45.6 ± 5.3	< 0.001*
AL‐PM systolic length (mm)	34.1 ± 5.0	33.4 ± 5.5	34.4 ± 4.81	0.092
PM‐PM diastolic length (mm)	37.0 ± 4.6	35.0 ± 5.2	37.9 ± 4.0	< 0.001*
PM‐PM systolic length (mm)	28.1 ± 4.4	27.6 ± 5.3	28.4 ± 4.0	0.142
Inter‐PM distance (mm)	23.6 ± 6.9	25.7 ± 8.0	22.7 ± 6.2	< 0.001*
PM‐LS (%)	23.6 ± 5.4	20.4 ± 6.1	24.9 ± 4.6	< 0.001*
AL‐PM‐LS (%)	23.2 ± 6.4	19.7 ± 6.8	24.7 ± 5.6	< 0.001*
PM‐PM‐LS (%)	24.0 ± 6.9	21.2 ± 7.9	25.2 ± 6.0	< 0.001*
Echocardiography data				
MR				
Mild (*n*, %)	228 (57.4)	63 (53.8)	165 (58.9)	0.35
Moderate (*n*, %)	98 (24.7)	31 (26.5)	67 (23.9)	0.589
Severe (*n*, %)	56 (14.1)	15 (12.8)	41 (14.6)	0.634
LVEDV (mL)	156 ± 71	172 ± 76	149 ± 68	0.006*
LVESV (mL)	83 ± 53	97 ± 58	77 ± 50	0.001*
LVSV (mL)	73 ± 29	76 ± 28	72 ± 29	0.25
LVEF (%)	50 ± 13	47 ± 13	51 ± 12	0.001*
LVEDD (mm)	56.0 ± 14.1	58.4 ± 12.8	54.9 ± 14.5	0.022*
LADD (mm)	45.7 ± 12.6	46.1 ± 11.3	45.5 ± 13.1	0.679

*Note:* *Indicates statistical significance.

Abbreviations: AL‐PM, anterolateral papillary muscle; BMI, body mass index; CABG, coronary artery bypass grafting; CHD, coronary heart disease; CK, creatine kinase; CK‐MB, creatine kinase‐MB; hcrp, high‐sensitivity C‐reactive protein; HCY, homocysteine; HDL, high‐density lipoprotein; HR, heart rate; hsTNT, high‐sensitivity troponin T; LADD, left atrial end‐diastolic dimension; LDH, lactic dehydrogenase; LDL, low‐density lipoprotein; LS, longitudinal strain; LVEDD, left ventricular end‐diastolic dimension; LVEDV/LVESV, left ventricular end‐diastolic/end‐systolic volume; LVEF, left ventricular ejection fraction; LVSV, left ventricular stroke volume; MR, mitral regurgitation; MV, mitral valve; myo, myoglobin; NT‐BNP, brain natriuretic peptide; NYHA, New York Heart Association; PCI, percutaneous coronary intervention; PM, papillary muscle; PMI, papillary muscle infarction; PM‐PM, posteromedial papillary muscle; SD, standard deviation; TIA, transient ischemic attack.

**Table 2:** Factors associated with PMI in all MR patients by univariate and multivariate logistic regression analyses.

Variables	Univariate analysis		Multivariate analysis	
	OR (95% CI)	*p*	OR (95% CI)	*p*
Sex (male)	2.239 (1.389–3.61)	0.001*	3.65 (1.881–7.081)	< 0.001*
Diabetes mellitus	2.884 (1.553–5.356)	0.001*	2.534 (1.13–5.68)	0.024*
LVEDV	1.004 (1.001–1.007)	0.004*	1.002 (0.996–1.007)	0.552
LVEF	0.972 (0.955–0.988)	0.001*	0.992 (0.964–1.02)	0.566
LVEDD	1.018 (1.002–1.034)	0.03*	1.003 (0.97–1.037)	0.873
AL‐PM diastolic length	0.857 (0.816–0.899)	< 0.001*	0.841 (0.77–0.92)	< 0.001*
PM‐PM diastolic length	0.86 (0.816–0.907)	< 0.001*	0.873 (0.79–0.964)	0.007*
Inter‐PM distance	1.063 (1.031–1.097)	< 0.001*	1.087 (1.028–1.15)	0.003*
AL‐PM‐LS (%)	0.872 (0.837–0.908)	< 0.001*	0.892 (0.843–0.943)	< 0.001*
PM‐PM‐LS (%)	0.917 (0.886–0.948)	< 0.001*	0.95 (0.91–0.992)	0.021*

*Note:* *Indicates statistical significance.

Abbreviations: AL‐PM, anterolateral papillary muscle; CI, confidence interval; LS, longitudinal strain; LVEDV, left ventricular end‐diastolic volume; LVEF, left ventricular ejection fraction; MR, mitral regurgitation; OR odds ratio; PM, papillary muscle; PMI, papillary muscle infarction; PM‐PM, posteromedial papillary muscle.

**Table 3:** Predictors of follow‐up arrhythmias with Cox regression analysis in MR patients.

Variables	Univariate analysis		Multivariate analysis	
	HR (95% CI)	*p*	HR (95% CI)	*p*
PMI	2.512 (1.687–3.742)	< 0.001*	1.644 (1.062–2.547)	0.026*
Age	1.012 (0.996–1.029)	0.134		
LVEDD	1.002 (0.99–1.015)	0.722		
LVEDV	1.0 (0.997–1.003)	0.96		
LVEF	0.985 (0.969–1.001)	0.062		
LADD	1.011 (1.0–1.022)	0.059		
AL‐PM‐LS (%)	0.908 (0.877–0.94)	< 0.001*	0.937 (0.903–0.973)	0.001*
PM‐PM‐LS (%)	0.913 (0.883–0.944)	< 0.001*	0.933 (0.902–0.965)	< 0.001*

*Note:* *Indicates statistical significance.

Abbreviations: AL‐PM, anterolateral papillary muscle; CI, confidence interval; HR, hazard ratio; LADD, left atrial end‐diastolic dimension; LS, longitudinal strain; LVEDD, left ventricular end‐diastolic dimension; LVEDV, left ventricular end‐diastolic volume; MR, mitral regurgitation; PM, papillary muscle; PM‐PM, posteromedial papillary muscle.

## 3 Results

Of the 397 MR patients (52.4 ± 13.9 years), 117/397 (29.5%) were assigned to the PMI group and 280/397 (70.5%) were assigned to the non‐PMI group. The interclass correlation analysis showed excellent agreement for PMI between the two observers (*κ* value 0.976). The mean age was 52.4 ± 14.3 years for patients with PMI and 52.3 ± 13.8 years for patients without PMI, which is not a significant difference (*p* > 0.05). Patient characteristics and medication are tabulated in Table 1. There was no significant difference between MR patients with and without PMI regarding BMI, HR, hypertension, alcohol, NYHA class (> III), history of TIA/infarction, partial laboratory findings (creatine kinase‐MB, high‐sensitivity C‐reactive protein, high‐density lipoprotein, low‐density lipoprotein), and medication (all *p* > 0.05). The PMI group had higher number of male patients (74.4% vs. 56.4%, *p* = 0.001) and higher number of patients with diabetes mellitus (20.5% vs. 8.2%, *p* = 0.001), coronary heart disease (CHD; 28.2% vs. 17.1%, *p* = 0.013), and partial laboratory findings (creatine kinase, myoglobin, lactic dehydrogenase, homocysteine, high‐sensitivity troponin T, and brain natriuretic peptide, all *p* < 0.05) than the non‐PMI group. The etiology of MR showed that 29 patients had rheumatic heart disease, 187 patients had degenerative heart disease, and 181 patients had ischemia/infarction‐related MR. The PMI group included higher number of patients with ischemia/infarction‐related MR (85.4% vs. 28.9%, *p* < 0.001) and the non‐PMI group showed higher number of patients with rheumatic heart disease (2.6% vs. 9.3%, *p* = 0.019) and degenerative heart disease (12% vs. 61.8%, *p* < 0.001). In PMI group, patients with rheumatic heart disease or degenerative heart disease were all accompanied by CHD. Among all cases, 17 patients underwent percutaneous coronary intervention (PCI), 10 patients underwent MV repair, 48 patients underwent MV replacement, 28 patients underwent aortic valve replacement, 31 patients underwent tricuspid valve repair, 2 patients underwent coronary artery bypass grafting, 11 patients underwent the Maze procedure, and others choose conservative treatment. In the PMI group, higher number of patients underwent PCI than the non‐PMI group (11.1% vs. 1.4%, *p* < 0.001); others showed no significant difference between the two groups (*p* > 0.05).

The distribution analysis of patients with PMI indicates a higher preference for the posterior PM (PM‐PM, 98/117, 83.8%), while AL‐PM is less frequently affected (45/117, 38.5%). There are 22 patients with combined PMI (22/117, 18.8%), which indicates that AL‐PM and PM‐PM are affected. PMI patients showed decreased AL‐PM diastolic length (41.5 ± 5.4 vs. 45.6 ± 5.3, *p* < 0.001), PM‐PM diastolic length (35.0 ± 5.2 vs. 37.9 ± 4.0, *p* < 0.001), PM‐LS (20.4 ± 6.1 vs. 24.9 ± 4.6, *p* < 0.001), AL‐PM‐LS (19.7 ± 6.8 vs. 24.7 ± 5.6, *p* < 0.001), PM‐PM‐LS (21.2 ± 7.9 vs. 25.2 ± 6.0, *p* < 0.001), and increased inter‐PM distance (25.7 ± 8.0 vs. 22.7 ± 6.2, *p* < 0.001). The AL‐PM and PM‐PM systolic length did not differ significantly between the two groups (*p* < 0.05), as well as the severity degree of MR (mild/moderate/severe) by echocardiography. Compared with non‐PMI patients, the PMI group showed increased LV end‐diastolic volume (LVEDV; 172 ± 76 vs. 149 ± 68, *p* = 0.004), LV end‐systolic volume (LVESV; 97 ± 58 vs. 77 ± 50, *p* = 0.001), larger LV end‐diastolic dimension (LVEDD; 58.4 ± 12.8 vs. 54.9 ± 14.5, *p* = 0.022), and decreased LV ejection fraction (LVEF; 47 ± 13 vs. 51 ± 12, *p* = 0.001), while the LV stroke volume (LVSV) and the left atrial end‐diastolic dimension (LADD) showed no significant difference (*p* > 0.05). In addition, PM‐PM‐LS (odds ratio [OR] = 0.893, 95% confidence interval [CI]: 0.833–0.957, *p* = 0.001) was found to be independently associated with PCI after adjusting for the potential confounders.

### 3.1 Reproducibility of PM Parameters

The AL‐PM diastolic/systolic length, PM‐PM diastolic/systolic length, and inter‐PM distance using CMR and LS parameters showed excellent and good intra‐ and interobserver reproducibility, respectively. For intraobserver reproducibility, the AL‐PM diastolic length had the highest reproducibility (ICC, 0.99, 95% CI: 0.98−0.99). For interobserver reproducibility, the inter‐PM distance had the highest reproducibility (ICC, 0.98, 95% CI: 0.98−0.99). The least reproducible segmental measurement for interobserver reproducibility was PM‐PM‐LS (ICC, 0.89, 95% CI: 0.87–0.91).

### 3.2 Factors Associated With PMI

Univariate logistic regression analyses indicated that male sex, diabetes mellitus, LVEDV, LVEF, LVEDD, AL‐PM, and PM‐PM diastolic length, inter‐PM distance, AL‐PM‐LS, and PM‐PM‐LS had significant associations with PMI in patients with MR in binary analyses. Multiple logistic regression analyses identified male sex (OR = 3.65, 95% CI: 1.881–7.081, *p* < 0.001], diabetes mellitus (OR = 2.534, 95% CI: 1.13–5.68, *p* = 0.024), AL‐PM diastolic length (OR = 0.841, 95% CI: 0.77–0.92, *p* < 0.001), PM‐PM diastolic length (OR = 0.873, 95% CI: 0.79–0.964, *p* = 0.007), inter‐PM distance (OR = 1.087, 95% CI: 1.028–1.15, *p* = 0.003), AL‐PM‐LS (OR = 0.892, 95% CI: 0.843–0.943, *p* < 0.001), and PM‐PM‐LS (OR = 0.95, 95% CI: 0.91–0.992, *p* = 0.021) to be independently associated with PMI after adjusting for the potential confounders (Table 2).

### 3.3 Follow‐Up Arrhythmia

**Figure 2:**
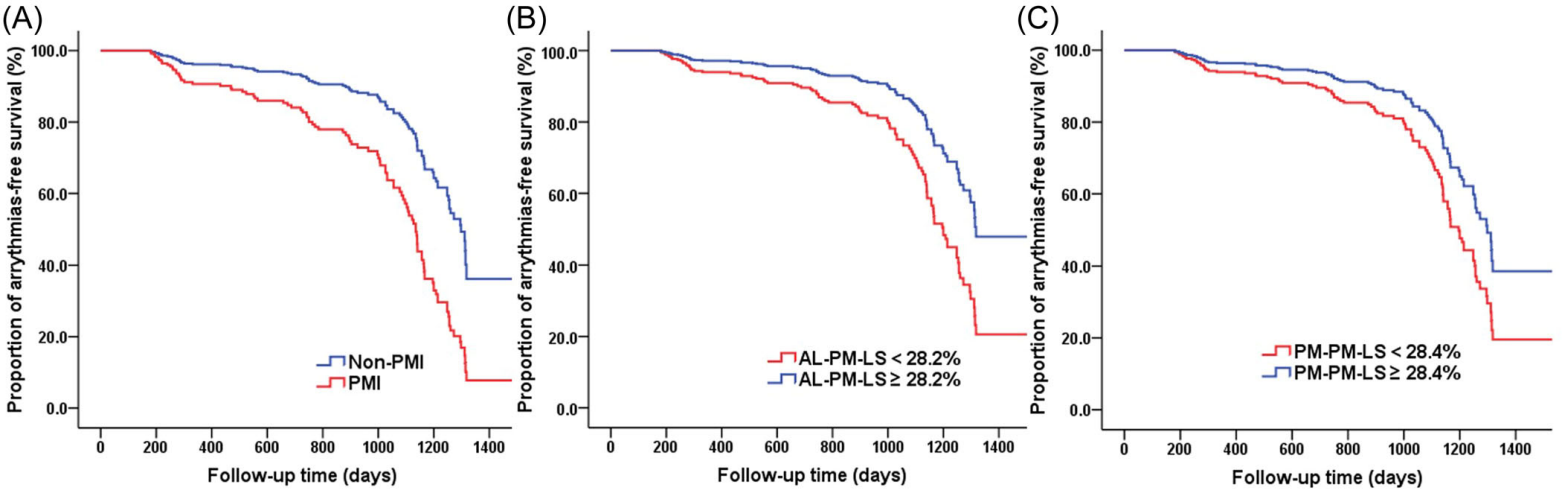
Proportion of follow‐up arrhythmia‐free survival. Follow‐up arrhythmia‐free survival curve plotted using the Kaplan–Meier method showed that patients with (A) PMI (log‐rank test, *p* < 0.001), (B) AL‐PM‐LS < 28.2% (log‐rank test, *p* = 0.011), and (C) PM‐PM‐LS < 28.4% (log‐rank test, *p* = 0.024) experienced a significantly higher incidence of arrhythmias during a mean follow‐up of (769 ± 367) days.

In total, 100 (100/397, 25.2%) patients had follow‐up arrhythmia, over a 769 ± 367‐day follow‐up. Using univariate Cox regression analyses, it was found that PMI, AL‐PM‐LS, and PM‐PM‐LS were univariate predictors of follow‐up arrhythmia in MR patients. Multivariate Cox regression analyses indicated that PMI (hazard ratio [HR] = 1.644, 95% CI: 1.062–2.547, *p* = 0.026), AL‐PM‐LS (HR = 0.937, 95% CI: 0.903–0.973, *p* = 0.001), and PM‐PM‐LS (HR = 0.933, 95% CI: 0.902–0.965, *p* < 0.001) remained significant (Table 3). The ROC analysis revealed that PMI, AL‐PM‐LS, and PM‐PM‐LS were associated with follow‐up arrhythmias, yielding AUCs of 0.631 (95% CI: 0.565–0.696, *p* < 0.001), 0.625 (95% CI: 0.589–0.715, *p* < 0.001), and 0.58 (95% CI: 0.512–0.647, *p* = 0.017), respectively, and the cutoff values for AL‐PM‐LS and PM‐PM‐LS were 28.2% and 28.4%, respectively. The Kaplan–Meier method illustrated that patients with PMI (log‐rank test, *p* < 0.001), AL‐PM‐LS < 28.2% (log‐rank test, *p* = 0.011), and PM‐PM‐LS < 28.4% (log‐rank test, *p* = 0.024) experienced a significantly higher incidence of arrhythmias (Figure 2).

## 4 Discussion

Compared with patients without PMI as indicated by MR, the PMI group had more male patients and there was higher prevalence of diabetes mellitus, a smoking history, CHD, larger LV volumes with impaired LV function, an increased inter‐PM distance, and a decreased AL/PM‐PM diastolic length, PM‐LS, and AL/PM‐PM‐LS. However, the severity degree of MR (mild, moderate, or severe) by echocardiography did not differ significantly. PMI patients with MR had a higher prevalence of PCI, and PM‐PM‐LS was independently associated with PCI. Multiple logistic regression analyses identified male sex, AL/PM‐PM diastolic length, inter‐PM distance, and AL/PM‐PM‐LS to be independently associated with PMI in patients with MR. Multivariate Cox regression analyses indicated that PMI and AL/PM‐PM‐LS are independently associated with follow‐up arrhythmia in patients with MR. Therefore, PMI is a significant imaging marker in patients with MR, and AL/PM‐PM‐LS is a notable value in predicting follow‐up arrhythmias.

PM contains approximately 13 chordae tendineae that are attached to the tips of both MV leaflets. Therefore, damage to one PM may affect both leaflets. AL‐PM has a dual blood supply (both from the left circumflex and the left anterior descending), whereas PM‐PM has a single blood supply (the right coronary artery or left circumflex) [18, 19]. Because of the vascular anatomy of the PM, PM‐PM was also more frequently affected. PMI had a reported frequency of 19%–32% in autopsy studies [4, 8], whereas in our study, it had a prevalence of 29.5%, and this supports these previous findings. Our study showed that PMI patients had more ischemia/infarction‐related MR, even the PMI patients with rheumatic/degenerative heart disease all had CHD, and PMI indicates a higher preference for PM‐PM than AL‐PM, indicating good agreement with previous studies [17, 18, 19]. In addition, PM‐PM‐LS was found to be independently associated with PCI, which indicated that PM‐PM‐LS was a very important imaging indicator; PMI patients with MR need more coronary artery revascularization to improve circulation.

PMI may lead to the development of MR by tethering the chordae and subsequent dysfunction of the PM–chorda–MV complex [19]. MR is believed to initiate myocardial remodeling associated with LV dilatation because of increased diastolic wall stress and decreased contractility with a consecutively increased LVESV [17]. A study by Grieve et al. [20] demonstrated that impaired LV function was an independent risk factor for the presence of PMI. Our study indicated that LV function was lower in the PMI group than in the non‐PMI group in patients with MR, especially those with significantly increased LVESV. Interestingly, our study showed that PMI was not associated with the severity of MR, but this result may have been demonstrated because most patients in the present study had moderate MR. Further study with a larger sample size should be carried out on patients with varying degrees of MR in the future.

Pambianchi et al.'s [4] study found that a lower PM‐LS was found in infarcted versus noninfarcted PM. Our findings showed that PMI with MR was mainly related to decreased AL‐PM/PM‐PM diastolic length, AL‐PM‐LS, and PM‐PM‐LS, indicating good agreement with previous studies. PM‐LS is a valid tool for assessing PM infarction, with the advantage of avoiding contrast media administration [4]. The presence, location, and extent of PMI depicted by LGE CMR were correlated with functional parameters.

PMI has been shown to have a negative prognostic value that could be related to ventricular arrhythmia [7, 8, 21]. Actually, PM is a source of ventricular arrhythmias in both structurally normal and abnormal hearts [22]. Furthermore, the PM parameter is strongly associated with arrhythmia after adjusting for the established clinical predictor in our study. The integration of PMI parameters and PM‐LS provides additional prognostic information regarding arrhythmia. PMI has also been shown to have a negative prognostic value that could be related to arrhythmias but may also be related to accompanying LV dysfunction and MR development [7, 8].

Our study has several limitations. This is a single‐center, retrospective, and observational cohort study with a relatively moderate sample size. There is no surgical or histopathological confirmation for the presence of PMI depicted by LGE MRI. However, as the published data provide evidence of excellent correlation of the LGE imaging of myocardial viability for the presence and extent of infarction with the histopathological results, this constitutes a minor limitation and justifies the assumption that LGE of PM represents ischemic necrosis [9, 23]. However, long‐term data are needed. Prospective, large multicenter studies are necessary to examine this obtained PM parameter in the selection of patients with MR undergoing aggressive medical therapy for further follow‐up data. Thus, further verification in a multicenter, prospective, and large‐sample study is required.

## 5 Conclusion

Our data contribute to an improved understanding of the clinical characteristics of PMI in patients with MR. The CMR‐derived PMI and PM‐LS are associated with arrhythmia and provide additive risk stratification for patients with MR. The analysis of PM‐LS improves the evaluation of PM dysfunction, and PM‐LS may be considered a valid tool for detecting PMI, because it has the advantages of avoiding gadolinium injection and being a quantitative approach.

## Ethics Statement

This study was approved by the local institutional review boards.

## Conflicts of Interest

The authors declare no conflicts of interest.

## Data Availability

The data underlying this article will be shared on reasonable request to the corresponding author.
